# Robots for surgeons? Surgeons for robots? Exploring the acceptance of robotic surgery in the light of attitudes and trust in robots

**DOI:** 10.1186/s40359-024-01529-8

**Published:** 2024-01-24

**Authors:** Balázs Szabó, Balázs Őrsi, Csilla Csukonyi

**Affiliations:** Institute of Psychology, University of Debrecen, Egyetem sugárút 1., 4032 Debrecen, Hungary

**Keywords:** Psychology, Medical robotics, Robotic surgical procedures, Human-robot interaction, Attitude

## Abstract

**Background:**

Over the last century, technological progress has been tremendous, and technological advancement is reflected in the development of medicine. This research assessed attitudes towards surgical robots and identified correlations with willingness to participate in robotic surgery based on factors influencing trust in automated systems.

**Method:**

Using data from a survey, which included the Multi-dimensional Robot Attitude Scale (MdRAS) and a questionnaire consisting of attitude statements regarding the factors affecting trust in automated systems, the experiment assessed the attitudes of healthcare workers and potential patients towards surgery robots, and attempted to find a correlation between these attitudes, age, and gender.

**Results and Conclusion:**

Statistical evaluation of the responses (*N* = 197) showed that positive attitude towards surgical robots showed a high correlation with the willingness to participate in robotic surgery and gave the strongest correlations with the MdRAS utility and negative attitude towards robots subscales. For the assessment of willingness, the MdRAS subscales alone did not provide a strong enough correlation. All factors examined showed a significant correlation with participation. Having faith in the surgery robot, the propensity to trust technology, the designer’s reputation, the ease of work that a surgical robot provides, positive experience with robots, and believing the surgeon is competent at operating the machine seemed to have been the most important positive correlations, while fear of errors gave the highest negative correlation. The healthcare workers and potential patients showed significant differences in the subscales of the questionnaire perceived risk and knowledge but no significant difference in the characteristics of the surgical robot. There was no difference in willingness to participate between the samples. Age did not show a significant correlation with the score achieved and willingness in any of the samples. Significant differences were found between male and female respondents, with men having more positive attitudes and being more likely to participate in surgeries using surgery robots than women. As a result, the research potentially sheds light on the factors that need to be considered when building trust in robotic surgery.

**Supplementary Information:**

The online version contains supplementary material available at 10.1186/s40359-024-01529-8.

## Background

There is an increasing focus on healthy human-robot interactions, healthcare, and social robots in the psychological field [1]. Robotics is a rapidly developing and therefore researched area, but its application in healthcare often faces obstacles [2, 3].

This limitation is not always technical [4]. Psychology may be the best tool for the widespread acceptance of robots and for exploring new potential uses for them [5]. Medical professionals experienced in robotic surgery emphasized psychological and social aspects when asked about the difficulties of working with a surgery robot [3].

Based on the aforementioned research, this study aims to shed light on people’s attitudes toward robotic surgery, their willingness to participate in surgeries using surgical robots, and their general attitudes toward robots using the Multi-dimensional Robot Attitude Scale (MdRAS) [6]. The sample can be disaggregated in terms of demographics and working in the healthcare field, which can provide insights into the differences and similarities in attitudes towards surgical robotics among the general population and healthcare workers.

The results could point the way towards building trust for healthcare workers and patients in surgical robotics, and improve attitudes towards surgical robots, which in turn could lead to more efficient medical work, lower anxiety from patients, better human-robot interaction, and opportunities for human-centered development of the surgical robot itself.

## Theoretical overview

### Attitude towards robots

Generally, trust is important from both the patient and the medical standpoint. Doctor-patient trust is related to patients’ perceived risk of medical treatment [7]. Trust in surgical robots shows the acceptance of robotic surgery while providing information about the treatment. Therefore, it is critical to understand attitudes towards automated systems and the role of trust in them. Moray and Inagaki [8] have emphasized in their definition of trust in automation the need to meet expectations and to be able to rely on automated systems to achieve the goal.

Cognitive processes play a prominent role in building trust in automated systems. Expectations about the machine’s capabilities best captured what people meant when they said they trusted machines [9]. Thus, the extent to which a robot is expected to be able to perform the task for which it is designed can have a decisive influence on its perceived reliability. The user must understand and be aware of the surgical robot’s competencies.

In addition to the cognitive processes, affective and behavioral components of trust perception are also relevant. People with more negative attitudes towards robots stay a greater physical distance away from the robot, or when the robot observes the subject, women keep a greater distance from the robot than men [10]. So, our attitudes and behavior towards an automated system are not only based on our knowledge and beliefs about it but also on how we feel about it. Frustration with faulty equipment strongly influences trust in the system, regardless of how much we know about its actual capabilities [11].

### Trust in automated systems and robots

Several definitions of trust have emerged within theories and research on the relationship between humans and automated systems. Moray and Inagaki [8] have highlighted in their definition of trust in automation the need to meet expectations and to be able to rely on automated systems to achieve a goal.

Trust in people is highly related to trust in automation [12]. Robots are often different in design, movement, and appearance from automated systems, and often perform their work at a distance, without an operator. These differences legitimately raise the question of whether there is a difference in the mechanisms by which humans build trust in automation and robots. The current literature suggests that similar factors influence trust and similar cognitive processes are involved in building trust in both cases [13–15], and the present research assumed this view.

### Factors affecting trust

There is extensive research on the factors involved in building trust in automated systems. The present study is based on Adams and colleagues’ paper: Trust in automated systems [16]. By reviewing three hundred relevant theoretical and research articles on the topic, they identified 22 factors that influence the development of trust in automated systems. The factors are divided into three groups: the properties of the automated system, the characteristics of the user, and the environment.

### The Da Vinci surgical system

The Da Vinci robotic surgical system is an increasingly evolving technology in medicine, used in general, head, neck, thoracic, cardiac, colorectal, urological, and gynecological surgeries for greater precision, lower risk, and faster recovery [17]. Physical discomfort and recovery time are also lower than in open and laparoscopic surgery [18].

## Method

The questionnaire for the survey consisted of three parts. The information and consent form was followed by a series of questions asking for demographic information (gender, age, education). Additional questions asked whether the person was working or studying in a medical field and, if they were about to undergo a medical procedure, whether they would consider themselves involved in a surgery involving a surgical robot on a Likert scale of one to seven.

**Fig. 1:**
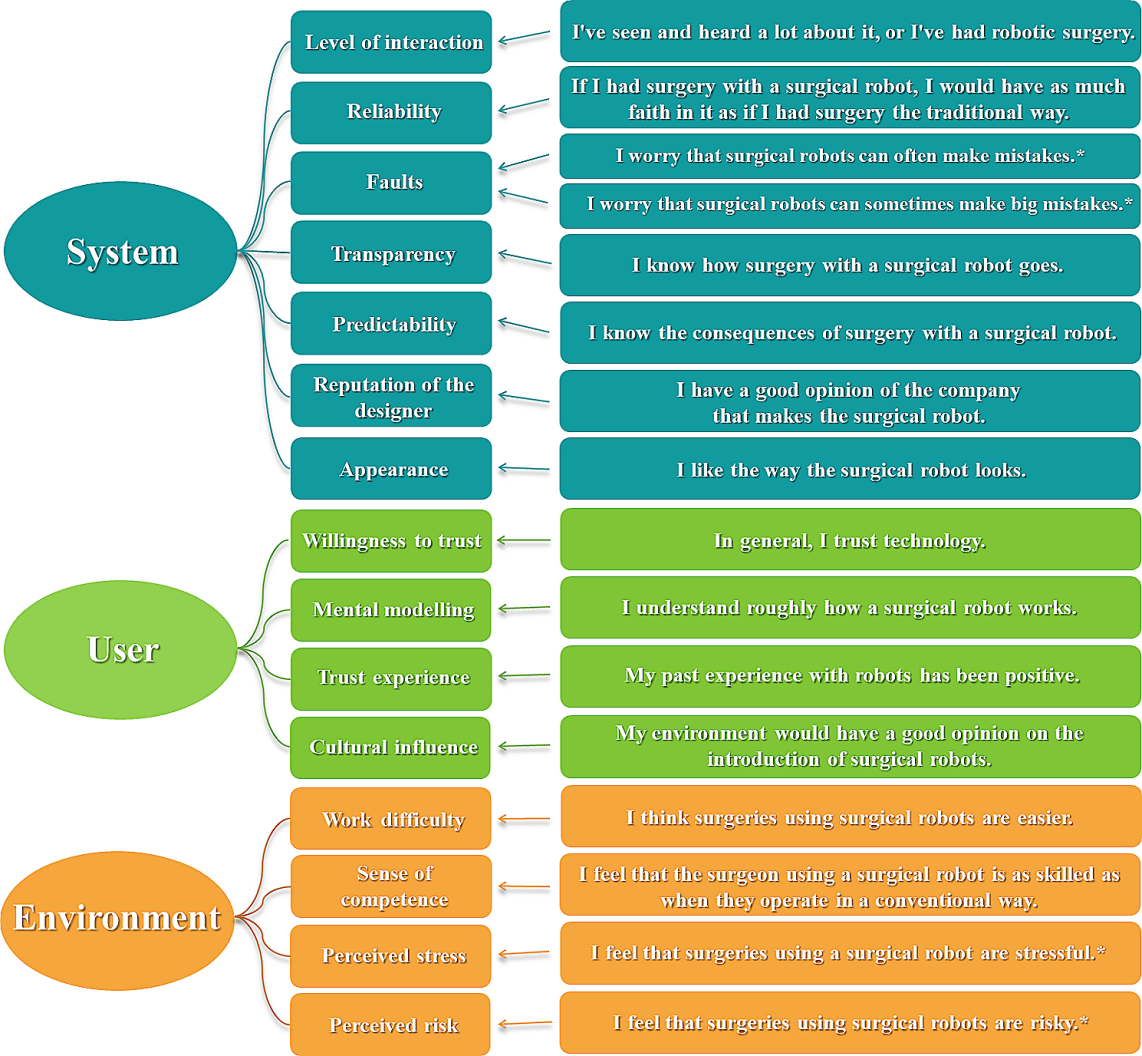
Trust factors and their associated questions. Note. Items marked with * require reverse scoring

As there was no specific surgical robot attitude questionnaire in the available literature at the time of conducting this research, the questionnaire developed for this study was based on the factors influencing trust in automated systems described previously. Sixteen questions were constructed for each of the three categories (Fig. 1). These items were also rated on a seven-point Likert scale (Supplement 1).

The Multi-dimensional Robot Attitude Scale, developed by Ninomiya et al. [6], measures twelve different dimensions of attitudes towards robots. These are trustworthiness, interest, negative attitude, self-efficacy, appearance, usefulness, cost, variety, control, peer support, operability, and environmental fit [19].

## Research findings

### Sample

A total of 197 people (143 women and 54 men) completed the questionnaire in online format. Their average age was 28.9 (SD = 12.4), the youngest respondent was 18, and the oldest was 87. In terms of education, 55 had completed university (27.9%), 17 colleges (8.6%), 97 were currently studying in higher education (49.2%), 8 of them had completed technical college (4.1%), 17 completed high school (8.6%), and 3 of them were currently studying in secondary school (1.5%). Of the subjects, 111 (56.3%) were not employed and 86 (43.7%) were working or studying in the health sector. In the healthcare sample, 35 (40.7%) were workers and 51 (59.3%) were students.

### Reliability and scales

The Cronbach’s alpha value of the questionnaire measuring attitudes towards surgical robots was too high (α = 0.915) with categories based on the literature. Transparency was highly correlated with several other items and was removed from the questionnaire. Due to the high correlation of “frequent errors” and “large errors” (r_S_=0.885), only the latter was retained, as it resulted in a lower α value, and was then labeled ‘errors’. The resulting Cronbach’s alpha value was 0.901, which, although exceeding the ideal value of 0.9, is more appropriate for the analysis of the results of this research.

The subscales based on the literature were changed after the exploratory factor analysis. The values are shown in Table 1.

**Table 1:** Subscales identified using exploratory factor analysis

	Factor						Uniqueness
	1		2		3		
Level of interaction					0.596		0.545
Predictability					0.908		0.219
Reliability	0.546						0.390
Errors			0.647				0.374
Reputation of the designer	0.737						0.409
Appearance	0.534						0.646
Willingness to trust	0.818						0.356
Mental modeling					0.584		0.465
Trust experience with machines	0.687						0.397
Cultural influence	0.496						0.556
Work difficulty	0.788						0.411
Sense of competence	0.729						0.502
Perceived stress			0.789				0.417
Perceived risk			0.897				0.198

The three new subscales are labeled such as:

1. Characteristics and reliability.
2. Perceived risk.
3. Knowledge of the surgical robot.

The interpretation of the second and third factors was quite simple. The second subscale included errors, perceived stress, and riskiness of surgery, all linked to the concept of “perceived risk”.

The third factor included the level of interaction with the surgical robot, predictability, and the mental model of the surgical robot. These could be connected to the heading ‘Knowledge of the surgical robot’.

The first factor included reliability, reputation of the designer, appearance, willingness to trust, trust experience with machines, cultural influence, difficulty of the work, and sense of skill, which were not linked to the other two subscales. The naming of this was not as straightforward: ‘Characteristics and reliability’.

### Descriptive statistics

In comparison to the average of the whole sample’s score on the surgical robot questionnaire (57.95), healthcare workers or students scored higher (61.08), and non-healthcare workers scored lower (55.53) out of the possible 98 points.

In terms of willingness to participate in the surgery, compared to the mean for the whole sample (4.79), healthcare workers or students scored higher (4.93), and non-healthcare workers scored lower (4.68). On average, both groups answered ‘rather yes’ to whether they would participate in a surgery where a surgical robot was used by a doctor (Tables 2 and 3).

**Table 2:** Descriptive statistics of the questionnaire, its subscales, and willingness-to-participate scores in the healthcare sample

	Surgical robot questionnaire	Characteristics and reliability	Perceived risk	Knowledge of the system	Willingness to participate
Mean	61.1	36.8	14.0	10.2	4.93
Median	60.5	36.0	14.0	9.00	5.00
SD	16.0	10.5	4.38	4.79	1.66
Minimum	21	8	3	3	1
Maximum	98	56	21	21	7
Shapiro-Wilk W	0.987	0.948	0.972	0.953	0.913
Shapiro-Wilk p	0.522	0.002	0.057	0.004	< 0.001

**Table 3:** Descriptive statistics of the questionnaire, its subscales, and willingness-to-participate scores in the non-healthcare sample

	Surgical robot questionnaire	Characteristics and reliability	Perceived risk	Knowledge of the system	Willingness to participate
Mean	55.5	35.0	12.4	8.14	4.68
Median	57	36	12	7	5
SD	15.3	9.68	4.43	4.20	1.84
Minimum	14	8	3	3	1
Maximum	92	56	21	21	7
Shapiro-Wilk W	0.966	0.912	0.973	0.928	0.906
Shapiro-Wilk p	0.006	< 0.001	0.023	< 0.001	< 0.001

### Hypothesis testing

#### H_1_

*The score on the surgical robot questionnaire and subscales correlates with the score on the MdRAS subscales*.

It is necessary to compare a new questionnaire to an already measured and validated psychological scale. Since normality is not met with the Shapiro-Wilk test for all but the score on the surgical robot questionnaire, the Spearman correlation and corresponding *p*-values are shown in Table 4, with significant values highlighted.

**Table 4:** Correlates of the MdRAS and the Surgical robot questionnaire

MdRAS subscales		Surgical robot questionnaire	Characteristics and reliability	Perceived risk	Knowledge of the system
Familiarity*	Spearman rho	0.335*	0.355*	0.185	0.184
	p	< 0.001	< 0.001	0.009	0.010
Interest*	Spearman rho	0.364*	0.384*	0.214	0.151
	p	< 0.001	< 0.001	0.002	0.034
Negative attitude*	Spearman rho	− 0.473*	− 0.452*	− 0.492*	− 0.188
	p	< 0.001	< 0.001	< 0.001	0.008
Self-efficacy*	Spearman rho	0.371*	0.326*	0.230	0.349*
	p	< 0.001	< 0.001	0.001	< 0.001
Appearance	Spearman rho	0.121	0.158	− 0.000	0.061
	p	0.090	0.027	0.996	0.398
Utility*	Spearman rho	0.503*	0.528*	0.277	0.289
	p	< 0.001	< 0.001	< 0.001	< 0.001
Cost	Spearman rho	− 0.175	− 0.124	− 0.217	− 0.167
	p	0.014	0.081	0.002	0.019
Variety	Spearman rho	0.197	0.220	0.106	0.081
	p	0.006	0.002	0.137	0.256
Control	Spearman rho	0.211	0.245	0.117	0.004
	p	0.003	< 0.001	0.102	0.959
Social support	Spearman rho	0.174	0.178	0.017	0.149
	p	0.014	0.012	0.817	0.037
Operation	Spearman rho	0.247	0.252	0.025	0.206
	p	< 0.001	< 0.001	0.727	0.004
Environmental fit	Spearman rho	− 0.160	− 0.141	− 0.204	− 0.047
	p	0.025	0.049	< 0.004	0.514

The results show that, although the correlation with most of the subscales is weak, utility (r_S_=0.503; *p* < 0.001) shows a medium positive correlation with the whole questionnaire and its first subscale, while negative attitude towards robots (r_S_=-0.473; *p* < 0.001) gives a similar medium but negative correlation with the entire questionnaire and its first and second subscales. In addition, it is worth mentioning the familiarity, interest, and self-efficacy subscales, as they stand out from the others in that they exceed the correlation value of 0.3.

These suggest that an individual’s negative attitudes towards robots will affect their attitudes towards surgical robots and that the degree to which they find robots useful will also affect surgical robots. Although not all subscales were correlated, there was a significant relationship, so the hypothesis was partially confirmed.

These two subscales also correlated the most with the willingness to participate in surgery using a surgical robot. A positive, moderate correlation was observed for utility (r_S_=0.490;*p* < 0.001), and a negative, weak correlation was observed for negative attitude (r_S_=-0.392;*p* < 0.01). Familiarity (r_S_=0.315;*p* < 0.001), interest (r_S_=0.328;*p* < 0.001), and self-efficacy (r_S_=0.307;*p* < 0.001) gave positive, weak correlations. These values are not strong enough predictors of willingness to participate in surgery, but they are worth mentioning as correlates. Not all subscales were correlated, but there was a significant relationship, so the hypothesis is partially confirmed.

#### H_2_

*Those who prefer surgery where a surgeon uses a surgical robot have a more positive attitude towards surgical robots.*

To examine the questionnaire as a predictor of willingness to participate in surgery with a surgical robot, the scores of the surgical robot attitude questionnaire were correlated with the respondents’ data on how much they would participate in surgery where a surgical robot is used by the doctor. The results are shown in Table 5.

**Table 5:** Correlation between willingness to participate in surgery using a surgical robot and the questionnaire

		Surgical robot questionnaire	Characteristics and reliability	Perceived risk	Knowledge of the system
Would you participate in a surgery where the doctor used a surgical robot?	Spearman rho	0.814	0.818	0.520	0.515
	p	< 0.001	< 0.001	< 0.001	< 0.001

The participation score shows a strong positive correlation with the questionnaire as a whole (r_S_=0.814; *p* < 0.001). Attitudes towards characteristics and reliability subscale show a similarly strong correlation (r_S_=0.818; *p* < 0.001), while perceived risk (r_S_=0.520; *p* < 0.001) and knowledge of surgical robot (r_S_=0.515; *p* < 0.001) subscales show a medium correlation. These results confirmed the hypothesis.

Table 6 shows how the factors correlate with willingness to participate in robotic surgery on an individual level. Every factor gave a statistically significant correlation, with reliability giving the highest positive (r_S_=0.781; *p* < 0.001). A general willingness to trust technology (r_S_=0.670; *p* < 0.001), the designer’s reputation (r_S_=0.598; *p* < 0.001), easing the workload (r_S_=0.594; *p* < 0.001), trust experience with machines (r_S_=0.558; *p* < 0.001) and the surgeon’s sense of competence (r_S_=0.534; *p* < 0.001) all gave moderate positive correlations. Cultural influence (r_S_=0.494; *p* < 0.001), having prior experience with the surgical robot (r_S_=0.488; *p* < 0.001), liking the appearance of the robot (r_S_=0.462; *p* < 0.001), understanding how it robot works (r_S_=0.430; *p* < 0.001), knowing the consequences of robotic surgery (r_S_=0.378; *p* < 0.001) all gave low positive correlation. Thinking that the surgical robot may make mistakes showed the highest negative (r_S_=-0.603; *p* < 0.001) correlation, followed by thinking these surgeries carry higher risks (r_S_=-0.432; *p* < 0.001). Thinking that surgeries using surgical robots are stressful (r_S_=-0.286; *p* < 0.001) gave the weakest, negligible correlation.

**Table 6:** Correlates of trust factors and willingness to participate in surgery using a surgical robot

Willingness to participate									
Level of interaction		Spearman rho		0.488	Mental modeling		Spearman rho		0.430
		p		< 0.001			p		< 0.001
Predictability		Spearman rho		0.378	Trust experience with machines		Spearman rho		0.558
		p		< 0.001			p		< 0.001
Reliability		Spearman rho		0.781	Cultural influence		Spearman rho		0.494
		p		< 0.001			p		< 0.001
Errors		Spearman rho		− 0.603	Work difficulty		Spearman rho		0.594
		p		< 0.001			p		< 0.001
Reputation of the designer		Spearman rho		0.598	Sense of competence		Spearman rho		0.534
		p		< 0.001			p		< 0.001
Appearance		Spearman rho		0.462	Perceived stress		Spearman rho		− 0.286
		p		< 0.001			p		< 0.001
Willingness to trust		Spearman rho		0.670	Perceived risk		Spearman rho		− 0.432
		p		< 0.001			p		< 0.001

#### H_3_

*Those who would prefer to participate in a surgery where a surgeon uses a surgical robot have more positive attitudes towards robots.*

To investigate which subscale of the MdRAS might be a good predictor of willingness to participate in surgery using a surgical robot. If the correlation is high for any of the subscales, it may be worthwhile in the future to add an attitude dimension with a high correlation to the questionnaire. The correlation between the score of participation in robotic surgery and the scores of the MdRAS subscales is shown in Table 7.

**Table 7:** Correlates of MdRAS and participation in surgery using a surgical robot

				Willingness to participate					
Familiarity		Spearman rho		0.275	Cost		Spearman rho		− 0.107
		p		< 0.001			p		0.135
Interest		Spearman rho		0.294	Variety		Spearman rho		0.242
		p		< 0.001			p		< 0.001
Negative attitude*		Spearman rho		− 0.395	Control		Spearman rho		0.230
		p		< 0.001			p		0.001
Self-efficacy		Spearman rho		0.289	Social support		Spearman rho		0.135
		p		< 0.001			p		0.058
Appearance		Spearman rho		0.077	Operation		Spearman rho		0.252
		p		0.285			p		< 0.001
Utility*		Spearman rho		0.471	Environmental fit		Spearman rho		− 0.049
		p		< 0.001			p		0.495

The strongest correlation was with the utility subscale, which showed a medium correlation with participation (r_S_=0.471; *p* < 0.001). The negative attitude towards robots subscale falls short of a medium negative correlation (r_S_=-0.395; *p* < 0.001), and the values for familiarity (r_S_=0.275; *p* < 0.001) and interest (r_S_=0.294; *p* < 0.001), although weak, are still correlated. Although not all subscales correlated, there was a significant relationship, so the hypothesis was partially confirmed.

#### H_4_

*There is no difference in attitudes towards surgical robots between healthcare workers and healthcare students.*

58% of the healthcare sample is made up of students in higher education in the medical field, so it is necessary to investigate the difference in the score of the surgical robot questionnaire compared to those who have worked in the healthcare field. To test the hypothesis, an independent sample t-test or Mann-Whitney U test with regards to normality (tested by Shapiro-Wilk test) was performed between the two groups on the scores on the surgical robot questionnaire. The results are shown in Table 8.

**Table 8:** Examination of the difference between the healthcare worker and student groups

	statistics	p
Surgical robot questionnaire	t=-0.025	0.980
Characteristics and reliability	U = 840	0.644
Perceived risk	U = 746	0.196
Knowledge of the system	U = 873	0.867

Note. H_a_ µ_working_ ≠ µ _studying_

There was no significant difference between respondents working in the healthcare field and those studying in the healthcare field, neither in terms of the questionnaire score (t=-0.025; *p* = 0.980) nor in terms of subscales. The obtained values confirm the hypothesis.

#### H_5_

*There is a difference between the healthcare and non-healthcare sample in the scores obtained on the surgical robot questionnaire.*

People in the healthcare sample will have more trust in surgical robots due to their professional proximity and education compared to those who know less about them. The results of the Mann-Whitney test between the two groups are shown in Table 9.

**Table 9:** Comparison of healthcare and non-healthcare workers’ scores on the Surgical robot questionnaire

		Mann-Whitney U	p
Surgical robot questionnaire		3936	0.035
Characteristics and reliability		4453	0.420
Perceived risk		3792	0.013
Knowledge of the system		3553	0.002

Note. H_a_ µ_healthcare_ ≠ µ _non−healthcare_

A comparison of the scores on the surgical robot attitude questionnaire using the Mann-Whitney test (*p* = 0.035) showed a significant difference between the scores of the two samples (mean_healthcare_=61.08, median_healthcare_=60.50; mean_non-healthcare_=55.53, median_non-healthcare_=57). In terms of subscales, only the subscale of characteristics and reliability does not show any difference between the groups (*p* = 0.420). There is a significant difference between the two samples, therefore the hypothesis is retained.

#### H_6_

*There is a difference between the healthcare and non-healthcare sample in their willingness to participate in robotic surgery.*

Using the Mann-Whitney U test, there is no significant difference between the two groups in the scores measuring the participation in a surgery where a surgeon uses a surgical robot (U = 4467; *p* = 0.433). It should be noted, that despite the non-significant difference, the mean of the healthcare sample scores on the question is higher (mean_healthcare_=4.93; mean_non-healthcare_=4.68), and their medians were the same (5). The hypothesis was rejected.

#### H_7_

*Older people trust surgical robots less.*

Age is a significant factor in the use of technology [20, 21]. Nowadays, there is a strong emphasis on the use of technology by older people and technological advances in this direction [22, 23]. However, it should be noted that even if age influences the amount of experience a person has with technology, if one is proficient in the use of technology, his or her attitude towards it will not differ due to age [24]. To establish the hypothesis, the correlation calculation between age and the surgery robot questionnaire score is shown in Table 10.

**Table 10:** Correlations with age

		Surgical robot questionnaire	Characteristics and reliability	Perceived risk	Knowledge of the system
Age	Spearman rho	0.095	0.035	0.150	0.101
	p	0.184	0.626	0.035	0.156

Table 8 shows a weak, non-significant correlation between age and the score on the surgical robot questionnaire in the sample (r_S_=0.095; *p* = 0.184). Among the subscales, perceived risk gave the strongest, although weak, but significant correlation (r_S_=0.150; *p* = 0.035). A non-significant, weak positive correlation (r_S_=0.151; *p* = 0.113) was observed for non-healthcare workers, and no correlation was observed for healthcare workers (r_S_=-0001; *p* = 0.992). As there was no significant correlation, the hypothesis was rejected.

#### H_8_

*Older people would be less likely to participate in robotic surgery.*

The correlation between age and the score of willingness to participate in surgery using a surgical robot resulted in a negligible correlation of participation with age (r_S_=0.031; *p* = 0.662). It was a weak negative in the healthcare sample (r_S_=-0.087; *p* = 0.427), and a weak positive correlation in the non-healthcare sample (r_S_=0.113; *p* = 0.239), with neither being significant. The hypothesis was not confirmed.

#### H_9_

*There is a difference between men and women in their attitude of trust towards surgical robots.*

There are numerous studies to identify gender differences in trust or technology use: men are more likely than women to give their trust [25], gender is a significant predictor of perceptions of conditional risks posed by technology, and women perceive risk more acutely than men [26]. To investigate whether men and women show differences in terms of their scores on the surgical robot questionnaire the scores were compared. The statistical values for the questionnaire scores of men and women for the two samples, as found by the Mann-Whitney test, are shown in Table 11.

**Table 11:** *P* values for comparing the scores of men and women

	Surgical robot questionnaire	Characteristics and reliability	Perceived risk	Knowledge of the system
Healthcare sample	0.026*	0.038*	0.253	0.132
Non-healthcare sample	0.019*	0.073	0.034*	0.066

In the healthcare sample, there was a significant difference between the scores on the questionnaire and the characteristics and reliability subscale (*p* < 0.05). In the non-healthcare sample, the scores on the surgical robot questionnaire and the perceived risk subscale showed a significant difference. These results suggest that men have more positive attitudes towards surgical robots, confirming the hypothesis. The mean and median of the questionnaire scores are shown in Table 12.

**Table 12:** Mean and median of the health and non-healthcare sample

	Gender	Mean	Median
Healthcare sample	female	59.5	58.0
	male	71.0	68.0
Non-healthcare sample	female	52.8	55.0
	male	60.0	62.0

#### H_10_

*There is a difference between men and women in willingness to participate in surgery using a surgical robot.*

Performing the Mann-Whitney test to test the hypothesis, there is a significant difference between genders. The statistical values are shown in Table 13.

**Table 13:** Statistical values of male and female participation willingness in surgery using a surgical robot

	Mann-Whitney U		p	gender	mean	median
Healthcare sample	255	0.016		female	4.77	5.00
				male	5.92	6.50
Non-healthcare sample	985	0.004		female	4.30	5.00
				male	5.29	6.00

Note: H_a_ µ_female_ ≠ µ _male_

Based on the sample median, men in both groups gave higher scores than women. Men in the healthcare sample gave a median response of “yes” to undergoing surgery, while women in this sample leaned towards a “rather yes” response. In the non-healthcare sample, these values were skewed towards the previous point, with men tending towards a “rather yes” response and women moving towards a “don’t know” response. These results confirm the hypothesis.

## Conclusion

**Fig. 2:**
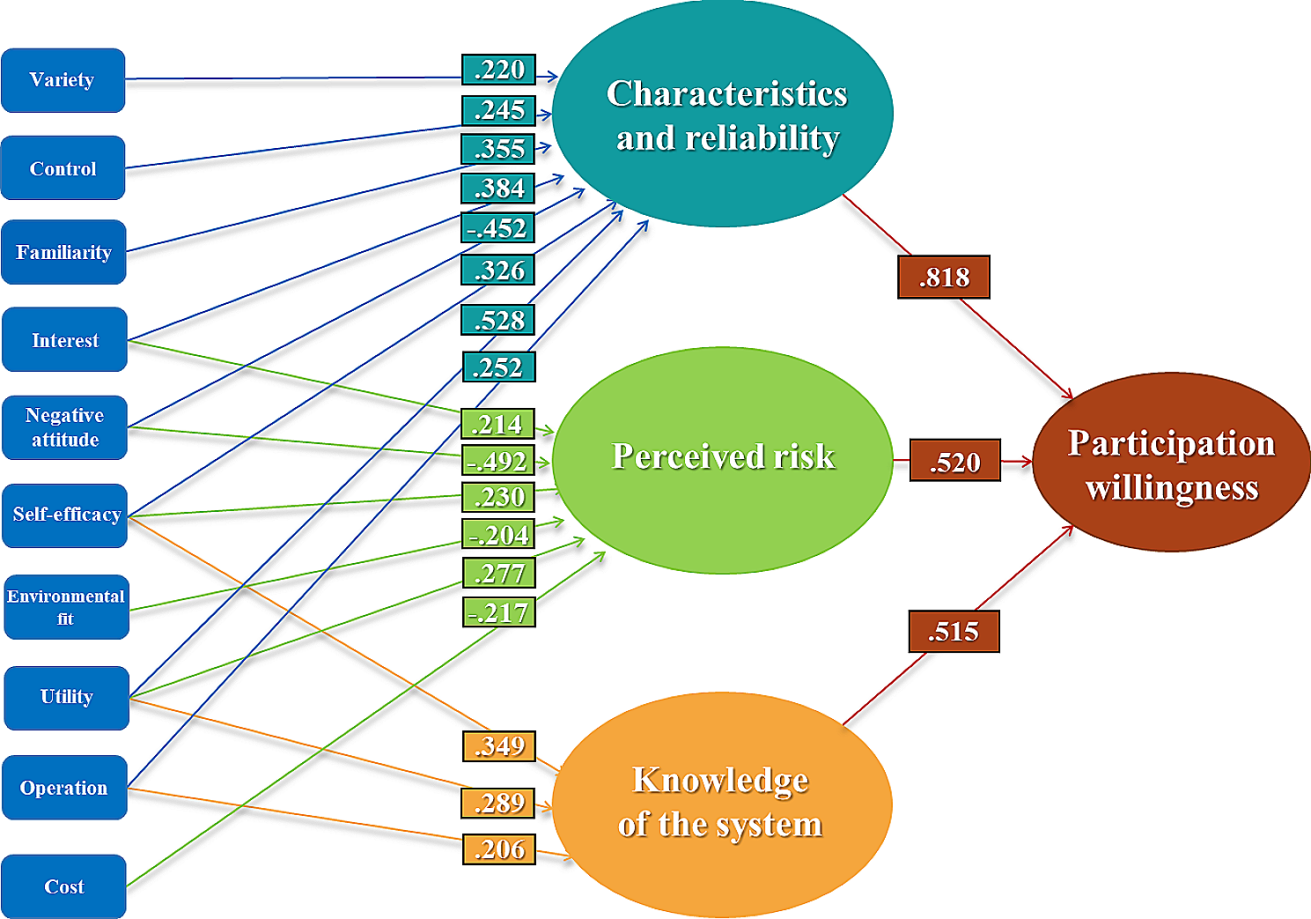
Correlation between MdRAS, the questionnaire, and participation willingness

Statistical evaluation of the responses from a total of 197 respondents showed that the attitude questionnaire on surgical robots did indeed show a high correlation with the willingness to participate in robotic surgery (Fig. 2). The questionnaire gave the highest correlations with the MdRAS utility and negative attitude towards robots subscales, with medium positive and negative correlations, respectively. For the assessment of willingness to participate, the MdRAS subscales alone did not provide a strong enough correlation.

**Fig. 3:**
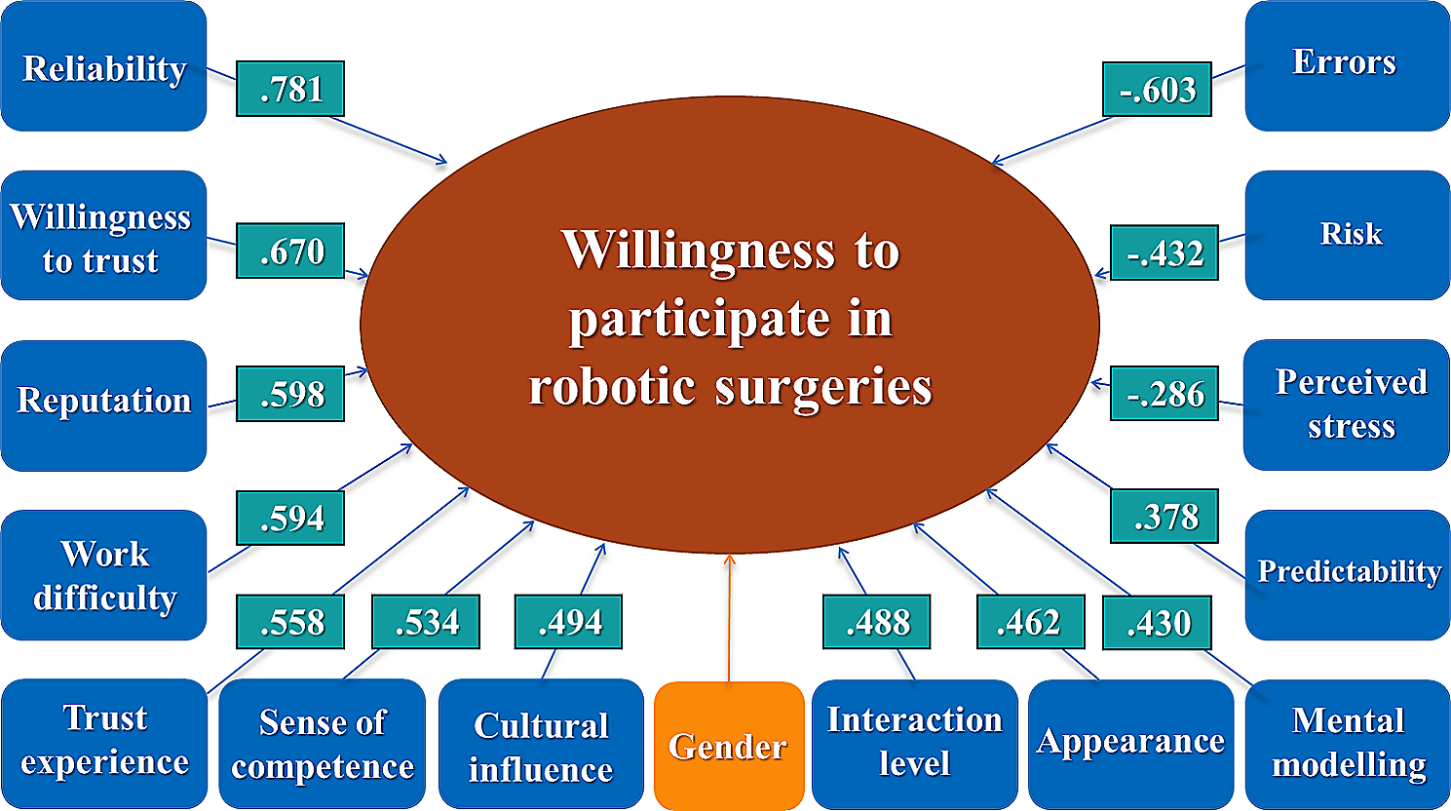
Factors influencing participation willingness

All factors examined showed a significant correlation with participation. Having faith in the surgery robot, willingness to trust technology, the designer’s reputation, the ease of work that a surgical robot provides, positive experience with robots, and believing the surgeon is competent at operating the machine seemed to have been the most positive correlations, and fear of errors gave the highest, moderate negative correlation. Thinking that surgeries using surgical robots are stressful was the only negligible correlation (Fig. 3).

When the sample was further subdivided, statistical analysis showed that it was not necessary to distinguish between the attitudes of healthcare workers and healthcare students toward surgical robots. The healthcare and non-healthcare samples showed significant differences in the subscales, perceived risk, and knowledge of the surgical robot, but no significant difference in the characteristics and reliability. The higher scores of healthcare workers can be explained by their broader knowledge of the risks associated with surgery and their familiarity with the robot due to their proximity to their profession. There was no difference in willingness to participate between the samples. Age did not show a significant correlation between the score achieved and willingness to participate in any of the samples. Significant differences were found between male and female respondents, with men having more positive attitudes and being more likely to participate in surgeries using surgery robots than women.

## Limitations, suggestions

It is worth mentioning that there is currently no consensus in the literature on exactly which factors influence the development of an individual’s trust in automated systems. Thus, attitudinal dimensions may be absent from the questionnaire that may be worth considering and measuring when replicating the research.

In addition, although the sample size was acceptable, the data collection was voluntary and online, which is not representative of society. 75% of respondents were between 18 and 31 years of age, so the survey will be able to make the most accurate findings for this group. Extended research (such as involving more subjects over the age of 30) should be conducted to generalize these results to other age groups. It should also be remarked, that to reduce the relatively high (0.901) Cronbach’s alpha, it is recommended that in the future the questionnaire be extended to include attitude dimensions not examined in the present research, or the existing items should be reformulated.

## Discussion

Interacting with technology is an increasingly essential part of everyday life, and trust seems to be an integrative factor. Robots and automated systems in healthcare are a significant part of robotics, with surgical robotic systems being a stepping stone, but the human side of it is still being explored.

The present research investigated people’s trust in and attitudes toward surgical robots by creating a questionnaire to measure attitudes towards surgical robots, exploring the attitudes of healthcare workers and potential patients towards the robots while finding significant differences between men’s and women’s attitudes.

Overall, the results of this study contribute to the exploration of people’s attitudes toward surgical robots, to the understanding of the human differences between conventional and robotic surgery, and to provide a basis for developing a more comprehensive attitude questionnaire towards surgical robots. It also sheds light on the factors necessary for the better adoption of robotic surgery, which could help healthcare professionals and surgical robot companies to better understand the preferences and needs of people during an intervention, which may contribute to the efficiency of healthcare.

As the reliance on automated systems in healthcare grows, it will be increasingly important to address attitudes and trust towards these systems, which may become more complex as robots advance. This research has hopefully laid another stone in the long road to better understanding the human side of automated systems.

### Electronic supplementary material

Below is the link to the electronic supplementary material.

Supplementary Material 1

## Data Availability

The dataset used and analyzed in the current study is available from the corresponding author upon reasonable request.

## References

[1] Henschel A, Laban G, Cross ES (2021). What makes a robot social? A review of social robots from science fiction to a home or hospital near you. Curr Rob Rep.

[2] Ouendi N, Hubaut R, Pelayo S, Anceaux F, Wallard L. The rehabilitation robot: factors influencing its use, advantages and limitations in clinical rehabilitation. Disabil Rehabilitation: Assist Technol, 2022;1–12. 10.1080/17483107.2022.2107095 35921160

[3] Randell R, Honey S, Alvarado N, Greenhalgh J, Hindmarsh J, Pearman A, Dowding D. Factors supporting and constraining the implementation of robot-assisted surgery: a realist interview study. BMJ open. 2019;9(6):e028635. 10.1136/bmjopen-2018-028635 PMC6589012 31203248

[4] Dzedzickis A, Subačiūtė-Žemaitienė J, Šutinys E, Samukaitė-Bubnienė U, Bučinskas V (2021). Advanced applications of industrial robotics: new trends and possibilities. Appl Sci.

[5] Sheridan TB (2020). A review of recent research in social robotics. Curr Opin Psychol.

[6] Ninomiya T, Fujita A, Suzuki D, Umemuro H. Development of the multi-dimensional robot attitude scale: Constructs of people’s attitudes towards domestic robots. In Social Robotics: 7th International Conference, ICSR 2015, Paris, France, October 26–30, 2015, Proceedings 7. 2015;482–491. Springer International Publishing.

[7] Wei D, Xu A, Wu X (2020). The mediating effect of trust on the relationship between doctor–patient communication and patients’ risk perception during treatment. PsyCh J.

[8] Moray N, Inagaki T (1999). Laboratory studies of trust between humans and machines in automated systems. Trans Inst Meas Control.

[9] Muir BM (1994). Trust in automation: part I. theoretical issues in the study of trust and human intervention in automated systems. Ergonomics.

[10] Takayama L, Pantofaru C. Influences on proxemic behaviors in human-robot interaction. In 2009 IEEE/RSJ International Conference on Intelligent Robots and Systems 2009;5495–5502. IEEE.

[11] Lee JD, Moray N (1992). Trust, control strategies and allocation of function in human-machine systems. Ergonomics.

[12] Hoffman RR, Johnson M, Bradshaw JM, Underbrink A (2013). Trust in automation. IEEE Intell Syst.

[13] Hancock PA, Billings DR, Schaefer KE, Chen JY, De Visser EJ, Parasuraman R (2011). A meta-analysis of factors affecting trust in human-robot interaction. Hum Factors.

[14] Lee JD, See KA (2004). Trust in automation: Designing for appropriate reliance. Hum Factors.

[15] Parasuraman R, Sheridan TB, Wickens CD (2008). Situation awareness, mental workload, and trust in automation: viable, empirically supported cognitive engineering constructs. J Cogn Eng Decis Mak.

[16] Adams BD, Bruyn LE, Houde S, Angelopoulos P, Iwasa-Madge K, McCann C. Trust in automated systems. Ministry of National Defence; 2003.

[17] Intuitive Surgical. (2021). Da Vinci Surgical Systems. https://www.intuitive.com/en-us/products-and-services/da-vinci.

[18] Wee IJY, Kuo LJ, Ngu JCY. A systematic review of the true benefit of robotic surgery: Ergonomics. Int J Med Rob Comput Assist Surg, 2020;16(4):e2113. 10.1002/rcs.2113 32304167

[19] Őrsi B, Lipták M, Csukonyi C (2020). A robotokkal kapcsolatos negatív attitűd- és szorongásmérő eszközök vizsgálata. ALKALMAZOTT PSZICHOLÓGIA.

[20] Czaja SJ, Charness N, Fisk AD, Hertzog C, Nair SN, Rogers WA, Sharit J (2006). Factors predicting the use of technology: findings from the Center for Research and Education on Aging and Technology Enhancement (CREATE). Psychol Aging.

[21] Volkom MV, Stapley JC, Amaturo V. Revisiting the digital divide: generational differences in technology use in everyday life. North Am J Psychol. 2014;16(3).

[22] Morris MG, Venkatesh V (2000). Age differences in technology adoption decisions: implications for a changing work force. Pers Psychol.

[23] Rogers WA, Fisk AD (2010). Toward a psychological science of advanced technology design for older adults. Journals of Gerontology Series B: Psychological Sciences and Social Sciences.

[24] Czaja SJ, Sharit J (1998). Age differences in attitudes toward computers. The Journals of Gerontology Series B: Psychological Sciences and Social Sciences.

[25] Buchan NR, Croson RT, Solnick S (2008). Trust and gender: an examination of behavior and beliefs in the investment game. J Econ Behav Organ.

[26] Siegrist M, Gutscher H, Earle TC (2005). Perception of risk: the influence of general trust, and general confidence. J Risk Res.

