# Supplementary material for: Robots for surgeons? Surgeons for robots? Exploring the acceptance of robotic surgery in the light of attitudes and trust in robots
Source: BMC Psychol. 2024 Jan 24;12:45. doi: 10.1186/s40359-024-01529-8 (PMC10807209; doi:10.1186/s40359-024-01529-8)
Supplement: Supplementary file 1 — Supplementary Material 1 [file 40359_2024_1529_MOESM1_ESM.docx]

##### Supplementary file.1

##### *Surgical Robot Trust Questionnaire*

Please indicate on a scale of 1 to 7 how true the following statements are for you, where:

1 – Strongly Disagree

2 – Disagree

3 – Slightly Disagree

4 – Don't know

5 – Slightly Agree

6 – Agree

7 – Strongly Agree

1. I've seen and heard a lot about it, or I've had robotic surgery.
2. If I had surgery with a surgical robot, I would have as much faith in it as if I had surgery the traditional way.
3. I worry that surgical robots can often make mistakes.*
4. I worry that surgical robots sometimes make big mistakes.*
5. I know how surgery with a surgical robot goes.
6. I know the consequences of surgery with a surgical robot.
7. I have a good opinion of the company that makes the surgical robot.
8. I like the way the surgical robot looks.
9. In general, I trust technology.
10. I understand roughly how a surgical robot works.
11. My past experience with robots has been positive.
12. My environment would have a good opinion on the introduction of surgical robots.
13. I think surgeries using surgical robots are easier.
14. I feel that the surgeon using a surgical robot is as skilled as when they operate in a conventional way.
15. I feel that surgeries using a surgical robot are stressful.*
16. I feel that surgeries using surgical robots are risky.*

*Note. Items marked with * require reverse scoring*
